# Public engagement in translational neuroscience: the good, the bad, and the ugly

**DOI:** 10.1093/braincomms/fcaf398

**Published:** 2025-11-04

**Authors:** Tara L Spires-Jones

**Affiliations:** Edinburgh, UK

## Abstract

**Graphical Abstract:**
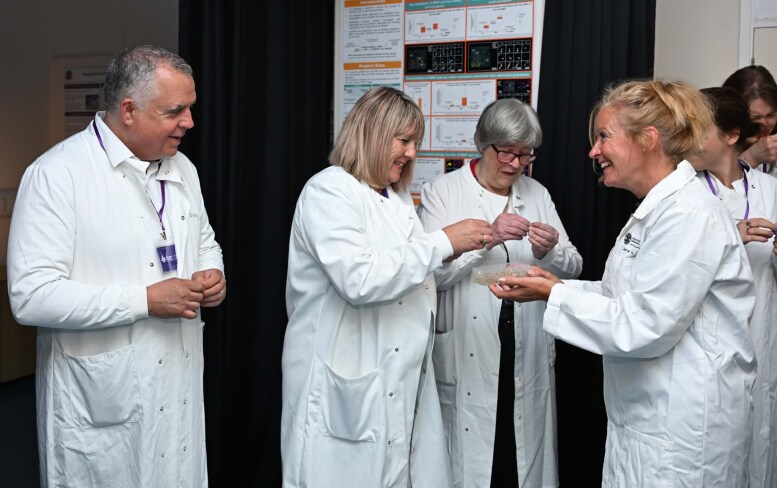

Welcome to Volume 7, Issue 6 of *Brain Communications*. One of the absolute pleasures of being Editor-in-Chief of our journal is the flush of pride when I hear one of our papers mentioned. As our reputation has grown, this has become more common, both in scientific talks and when listening to the news. Several of our papers have received wide media attention this year, including Patel and colleagues’ work examining multimorbidity and dementia risk in UK BioBank data^1^ and Gonzalez-Ortiz and colleagues’ study finding high phospho-tau 217 levels in blood samples from newborns.^2^ Since we are often funded by public money and charities, I think sharing results with the public is a responsibility of translational neuroscientists. This is not always easy. Institutional press offices are often not interested in promoting papers unless they are directly relevant to people—for example, new treatments. To make more fundamental work interesting, there is temptation among journalists and press officers to over-interpret results. In the UK, the Science Media Centre does a fantastic job of curating comments from experts in the field on studies being covered in the news.^3^ I regularly comment on dementia research studies for the Science Media Centre, providing independent context for news stories about new findings.

I find working with the Science Media Centre extremely rewarding, although there are some disadvantages to media engagement. When I began commenting, it was quite intimidating to critique other people’s papers publicly, but for most comments, I can honestly say that the paper is interesting and important, but point out important limitations and temper public expectations of the outcomes of the research. At first, I was also worried that other scientists might see this engagement as ‘attention seeking’ or less than serious scientific behaviour. Over the years, most feedback I have received has been positive, and, frankly, as I’ve gotten older I have stopped caring as much about what others think. After commenting on a paper for the Science Media Centre, I am often contacted for media interviews about the research, which is fun but requires more preparation than a short comment on the paper, and it is intimidating to be interviewed live. Media engagement also moves at a completely different timescale to normal scientific work. Everything moves fast, there is not much advance warning, and things can be cancelled at the last minute if a more interesting story comes up.

Another disadvantage of media engagement is that even with carefully crafted press releases and cautious interviews, there can be unpleasant misunderstandings. Our group recently published a paper, in collaboration with veterinarian colleagues, looking at pathology in the brains of cats with cognitive dysfunction syndrome, which is similar to human dementia.^4^ Our press office wanted to issue a press release on the paper as they quite rightly thought there would be wide interest in a story about ‘cats with dementia’. We released a careful comment which was widely picked up in the press. While most responses were positive, the lead author, Dr Robert McGeachan, who had done media interviews about the study, received messages accusing us of performing experiments on living cats. This was not at all the case. These were much loved pets receiving excellent care, whose owners agreed to donate brain tissue after they died. Despite the few negative comments, overall we had overwhelmingly positive interest in our work. For me, the opportunity to raise the profile of the importance of scientific research through media engagement far outweighs the rare negative responses.

Another very rewarding type of engagement for our group of dementia researchers comes from our interactions with people living with diseases that cause dementia. We regularly host visits with Alzheimer Scotland to bring people into our centre for tours of the laboratories and interactions with scientists (see Fig. 1, for example). Over a cup of tea and a nice piece of cake, people share their stories of dementia with us, and we have the chance to ask what they think of our research and what questions and problems are important to them. This brings important perspective to our work and provides trainees with experience explaining our complex fundamental science to people who will likely not directly benefit themselves, but who derive hope from our efforts that aim to help people with dementia in the future.

**Figure 1:**
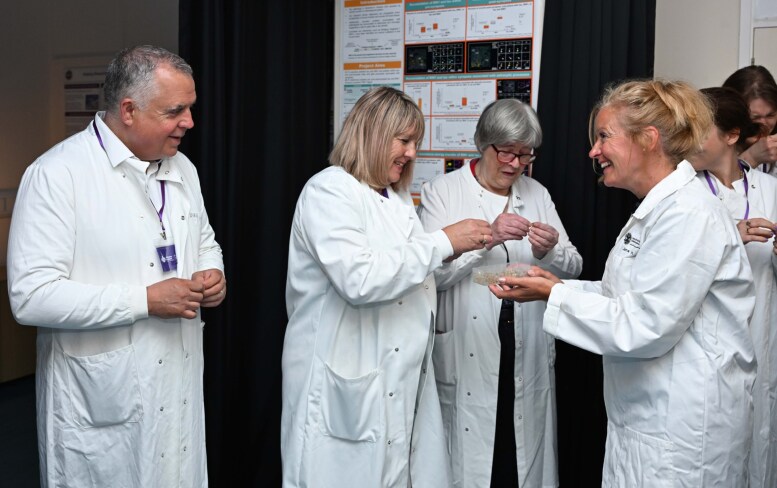
A visit by Alzheimer Scotland charity workers and members of their Active Voice group of people living with dementia to the University of Edinburgh Centre for Discovery Brain Sciences.

I think that my experience engaging with the media and members of the general public about research has made me a better scientist. I’ve certainly read more papers carefully outside of my direct area of interest than I would have otherwise, learning more broadly about current research. My grant writing has improved, particularly the ability to put our work into the wider context of the field. Experiences of talking about dementia to public audiences inspired me to write a book on advances in dementia research, which should be published in 2026. I’m not sure whether anyone other than my mother will read it, but the experience of distilling current research and speculating about future treatments and preventions for a general audience has been a rewarding experience.

For *Brain Communications* authors, if you want to embargo the publication of your papers with us to coordinate a press release with your institution, let us know, we can help with that!

The cover image for this issue comes from Jung *et al*.,^5^ with artwork by William Sommer, and depicts a helmet overlaid with brain anatomy to highlight their work on sulcal morphology as a potential biomarker of repetitive head impacts and chronic traumatic encephalopathy risk.

## References

[1] Patel R, Gillis G, Mackay CE, et al The lifetime accumulation of multimorbidity and its influence on dementia risk: A UK biobank study. Brain Commun. 2025;7(4):fcaf222. 40672935 10.1093/braincomms/fcaf222 PMC12266833

[2] Gonzalez-Ortiz F, Vávra J, Payne E, et al The potential dual role of tau phosphorylation: Plasma phosphorylated-tau217 in newborns and Alzheimer’s disease. Brain Commun. 2025;7(3):fcaf221. 40574977 10.1093/braincomms/fcaf221 PMC12198956

[3] Science Media Centre . Accessed October 2, 2025. https://www.sciencemediacentre.org/

[4] McGeachan RI, Ewbank L, Watt M, et al Amyloid-Beta pathology increases synaptic engulfment by Glia in feline cognitive dysfunction syndrome: A naturally occurring model of Alzheimer’s disease. Eur J Neurosci. 2025;62(3):e70180. 40790741 10.1111/ejn.70180 PMC12340200

[5] Jung LB, Mirmajlesi AS, Stearns J, et al Sulcal morphology in former American football players. Brain Commun. 2025;7(5):fcaf345. 41048544 10.1093/braincomms/fcaf345 PMC12492488

